# The many faces of nodal and splenic marginal zone lymphomas. A report of the 2022 EA4HP/SH lymphoma workshop

**DOI:** 10.1007/s00428-023-03633-3

**Published:** 2023-09-01

**Authors:** Alberto Zamò, Michiel van den Brand, Fina Climent, Laurence de Leval, Stefan Dirnhofer, Lorenzo Leoncini, Siok-Bian Ng, Sarah L. Ondrejka, Leticia Quintanilla-Martinez, Lorinda Soma, Andrew Wotherspoon

**Affiliations:** 1 Institute of Pathology, University of Würzburg, Josef-Schneider-Str. 2, 97080 Würzburg, Germany; 2 Pathology-DNA, Location Rijnstate Hospital, Wagnerlaan 55, 6815AD, Arnhem, The Netherlands; 3 Department of Pathology, Radboud University Medical Center, Nijmegen, The Netherlands; 4 Department of Pathology, Hospital Universitari de Bellvitge-IDIBELL, L’Hospitalet de Llobregat, Barcelona, Spain; 5 Department of Laboratory Medicine and Pathology, Institute of Pathology, Lausanne University Hospital and Lausanne University, Lausanne, Switzerland; 6 Institute of Medical Genetics and Pathology, University Hospital Basel, University of Basel, Basel, Switzerland; 7 Department of Medical Biotechnology, Section of Pathology, University of Siena, Siena, Italy; 8 Department of Pathology, Yong Loo Lin School of Medicine, National University of Singapore, Singapore, Singapore; 9 Cancer Science Institute of Singapore, National University of Singapore, Singapore, Singapore; 10 Pathology and Laboratory Medicine Institute, Cleveland Clinic, Cleveland, OH USA; 11 Institute of Pathology and Neuropathology, Eberhard Karls University of Tübingen and Comprehensive Cancer Center, University Hospital Tübingen, Tübingen, Germany; 12 Department of Pathology, City of Hope Medical Center, Duarte, CA USA; 13 Department of Histopathology, Royal Marsden Hospital, London, UK

**Keywords:** Marginal zone lymphoma, Splenic marginal zone lymphoma, Splenic diffuse red pulp small B-cell lymphoma, Marginal zone lymphoma transformation, Pediatric nodal marginal zone lymphoma, Pediatric nodal marginal zone hyperplasia

## Abstract

Session 3 of the lymphoma workshop of the XXI joint meeting of the European Association for Haematopathology and the Society for Hematopathology took place in Florence, Italy, on September 22, 2022. The topics of this session were splenic and nodal marginal zone lymphomas, transformation in marginal zone lymphomas, and pediatric nodal marginal zone lymphomas and their differential diagnosis as well as related entities. Forty-two cases in these categories were submitted to the workshop, including splenic lymphomas (marginal zone and diffuse red pulp lymphomas), transformed marginal zone lymphomas (splenic and nodal), nodal marginal zone lymphomas with increased TFH-cells, and pediatric nodal marginal zone lymphomas. The case review highlighted some of the principal problems in the diagnosis of marginal zone lymphomas, including the difficulties in the distinction between splenic marginal zone lymphoma, splenic diffuse red pulp lymphoma, and hairy cell leukemia variant/splenic B-cell lymphoma with prominent nucleoli which requires integration of clinical features, immunophenotype, and morphology in blood, bone marrow, and spleen; cases of marginal zone lymphoma with markedly increased TFH-cells, simulating a T-cell lymphoma, where molecular studies (clonality and mutation detection) can help to establish the final diagnosis; the criteria for transformation of marginal zone lymphomas, which are still unclear and might require the integration of morphological and molecular data; the concept of an overlapping spectrum between pediatric nodal marginal zone lymphoma and pediatric-type follicular lymphoma; and the distinction between pediatric nodal marginal zone lymphoma and “atypical” marginal zone hyperplasia, where molecular studies are mandatory to correctly classify cases.

**Supplementary Information:**

The online version contains supplementary material available at 10.1007/s00428-023-03633-3.

## Introduction

This paper summarizes the findings of the lymphoma workshop session (session 3), dedicated to splenic and nodal marginal zone lymphoma (SMZL and NMZL), during the XXI meeting of the European Association for Haematopathology (EAHP) held in Florence in September 2022. Due to the heterogeneity of marginal zone lymphomas (MZL), a broad range of entities and diagnostic scenarios were discussed during this session. Forty-two cases were submitted. These were grouped according to unifying themes which included SMZL and its differential diagnosis, the diagnosis of transformation in MZL, T follicular helper (TFH) cell hyperplasia in MZL, and pediatric nodal MZL (PNMZL) and related entities. This review summarizes the findings of the cases submitted for this workshop session to provide an overview of current diagnostic criteria, strategies, and pitfalls.

## Splenic marginal zone lymphoma and its differential diagnosis

### Splenic marginal zone lymphoma

SMZL is the most common type of splenic B-cell lymphoma. It is an indolent B-cell lymphoma involving the spleen and usually also the peripheral blood and bone marrow. Patients present with splenomegaly and often enlarged splenic hilar lymph nodes, but without peripheral lymphadenopathy. In the spleen, the white pulp is markedly expanded and replaced by nodular aggregates of neoplastic B-cells, commonly showing a biphasic pattern with central small B-cells surrounded by a rim of cells with small to medium sized nuclei with more abundant cytoplasm. Some of the nodules might contain a residual reactive germinal center. In addition, the red pulp is also infiltrated by small neoplastic B-cells. Lymphoma cells in the peripheral blood usually show short polar villi (so-called villous lymphocytes) although this feature is not strictly required for the diagnosis and is not completely specific as other splenic lymphomas can have villous appearing lymphocytes. Histological examination of spleen tissue is considered the gold standard for a correct diagnosis but diagnostic splenectomies have become much less common and splenic biopsies are only rarely performed. Therefore, the diagnosis often depends on examination of a bone marrow biopsy where SMZL shows a nodular, interstitial, and intrasinusoidal pattern of infiltration. Since this pattern is not entirely specific, a precise differential diagnosis between SMZL and other indolent splenic B-cell lymphomas (such as splenic diffuse red pulp B-cell lymphoma) might not be possible on bone marrow alone. It is still unclear whether this distinction is of clinical importance. The diagnostic category of SMZL remained unchanged between the revised 4th edition and the 5th edition of the World Health Organization (WHO) classification [1] and also in the International Consensus classification (ICC) [2, 3].

Immunophenotypically SMZL shows a mature B-cell phenotype without expression of germinal center markers or cyclin D1. Expression of CD5 is present in 19–25% of cases (by flow cytometry) and is associated with higher lymphocytosis and diffuse bone marrow infiltration [4, 5]; less than 1% of SMZL display a *CDK6* translocation and more frequent expression of CD5 (55%) as well as an increase in cells resembling prolymphocytes [6]. Chronic lymphocytic leukemia (CLL/SLL) and mantle cell lymphoma (MCL) should be excluded in these cases; helpful markers include LEF1 (normally positive in B-CLL/SLL) and TCL1 (SMZL is mostly negative, in contrast to CLL/SLL and MCL, both of which are normally TCL1-positive) [7] while staining for SOX11 might be of limited values, splenic MCL is often negative [8]. Although the sensitivity varies between studies from 24 to 100%, detection of MNDA expression is also a useful tool since it is one of the few “positive” markers in SMZL (and in other MZL) [7, 9–11] that can be useful in the distinction from follicular lymphoma (almost always negative) and lymphoplasmocytic lymphoma (LPL, frequently negative) [12]. By flow cytometry, CD11c is usually positive but with a lower intensity than in other splenic B-cell lymphomas. Staining for CD200 is positive but with a lower intensity than in hairy cell leukemia [13]. CD25 is commonly negative while CD103 and CD123 are typically negative [14]. Annexin A1 is consistently negative [15].

Excluding the extremely rare *CDK6* translocation, no recurrent chromosomal rearrangements have been identified in SMZL. Deletion of chromosome 7q is the most frequent cytogenetic abnormality and is detected in approximately 40% of SMZLs [16] and is quite specific for this entity [17]. Less frequent alterations include gains of 3q, 8q, 9q, 12q, and 18q, and loss of 6q and 8p, 14q, and 17p [16–20].

Studies into the mutational landscape of SMZL have identified recurrent mutations in *KLF2* and genes involved in NOTCH and NF-kappaB signaling pathways. KLF2 is a transcription factor involved in cell survival, NF-kappaB signaling, and cell trafficking [21]. *KLF2* mutations are detected in 20–40% of SMZLs and cause displacement of the KLF2 protein to the cytoplasm preventing it from inhibiting NF-kappaB activation [22–27]. Mutations of genes involving the NOTCH pathway (i.e. *NOTCH1*, *NOTCH2*, *SPEN*, *FBXW7*, *DTX1*) are detected in 40% of SMZL with *NOTCH2* being most frequently involved [21, 24, 26, 28–31]. Mutations in genes involved in NF-kappaB signaling are found in approximately one-third of SMZLs and include mutations in *TNFAIP3*, *IKBKB*, *TRAF3*, *CARD11*, and *BIRC3* [22–24, 29, 32–34]. Mutations in *MYD88* have been reported in 8% of SMZL with approximately two-thirds representing the classical L265P mutation [23, 30, 35].

*TP53* mutations are found in approximately 15% of SMZLs and are associated with a worse outcome [23, 24, 36]. A recent study, which aimed to integrate the molecular findings, detected two prominent molecular clusters in SMZL; the NNK cluster represented 58% of SMZLs and contained mutations in NF-kappaB, NOTCH, and KLF2 modules, while the DMT cluster represented 32% of SMZLs and was associated with mutations in genes involved in DNA damage response, MAPK, and TLR modules [32, 37]. In this work, the NNK cluster was associated with a worse survival. Some earlier reports also found a worse outcome in cases with *NOTCH* mutations, although this was not confirmed in all studies [24, 28, 38].

**Fig. 1:**
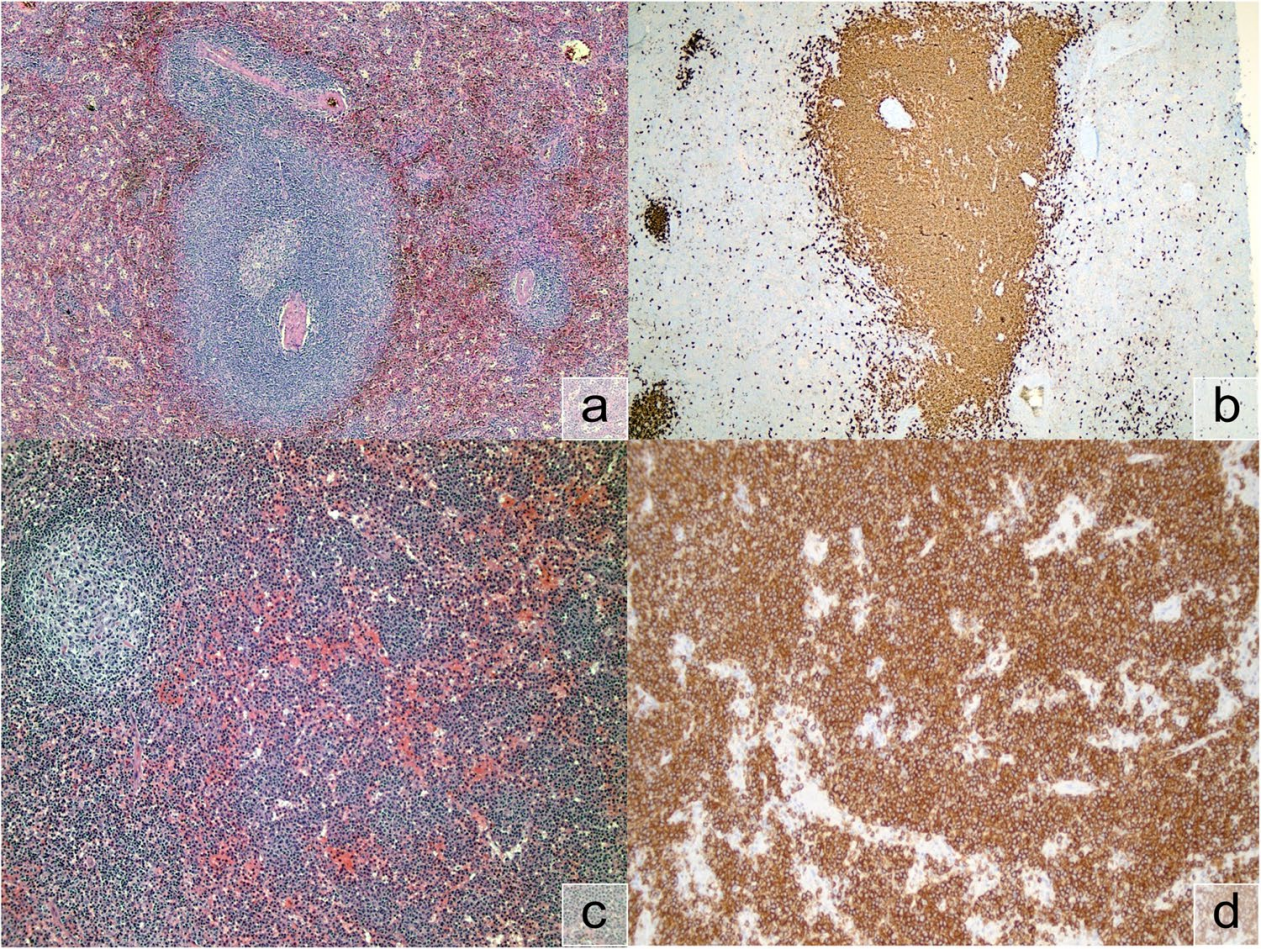
Splenic marginal zone lymphoma (SMZL) and splenic diffuse red pulp lymphoma (SDRPL); **a** SMZL, H&E (LYWS-1052). **b** SMZL, CD20 (LYWS-1052). **c** SDRPL, H&E (LYWS-1150). **d** SDRPL, CD20 (LYWS-1150)

One submitted case of SMZL (LYWS-1052 by Sohaib M. Al-Khatib) showed the prototypical morphology of SMZL in the spleen (Fig. 1a and 1b). In the SMZL case submitted by Gabriel Caponetti (LYWS-1165), sequencing analysis demonstrated a typical but relatively uncommon molecular constellation with mutations in *BIRC3* and *TP53*. Mutations in *BIRC3* are typical but relatively rare (~11%) in SMZL and might predict resistance to Ibrutinib therapy [39].

The distinction of SMZL from other types of low-grade B-cell lymphoma can be problematic since no specific marker is available and the morphological differences can be very subtle. Distinction from other MZLs depends on the clinical presentation and the location of the disease. Follicular lymphoma (FL), MCL, and CLL are usually easily distinguished based on morphology and immunohistochemistry. Distinction from splenic diffuse red pulp small B-cell lymphoma (SDRPL) and splenic B-cell lymphoma/leukemia with prominent nucleoli (SBLPN)/hairy cell leukemia variant (HCLv) can be more difficult and is discussed in more detail below (Table 1).

**Table 1:** Differential diagnosis of splenic B-cell lymphomas with predominant infiltration of the spleen

	SMZL	SDRPL	SBLPN/HCLv	HCL
Usual clinical presentation	Splenomegaly, variable lymphocytosis, cytopenia in 25% of patients, rarely B-symptoms	Splenomegaly, low to moderate lymphocytosis, no or mild cytopenia, no monocytopenia, rarely B-symptoms	Splenomegaly, lymphocytosis, anemia and thrombocytopenia sometimes, no monocytopenia	Splenomegaly, lymphocytosis often not prominent, prominent cytopenia with monocytopenia
Peripheral blood cytology	Polymorphic cells, small cells and lymphoplasmacytoid, thin and short villi with uneven distribution in a small proportion of cells	Monomorpic small to medium sized cells. Frequent small, broad-based villi	Medium to large monomorphic cells, prominent nucleoli, basophilic cytoplasm, fine villi	Small to medium sized monomorphic cells with indented nuclei with inconspicuous nucleoli and abundant long villi
Spleen histology	Nodular expansion in white pulp with biphasic or monophasic pattern, also red pulp involvement	Diffuse red pulp involvement, white pulp effacement	Diffuse red pulp involvement, white pulp effacement	Diffuse red pulp involvement, white pulp effacement
Bone marrow histology	Nodular and intrasinusoidal, sometimes interstitial	Intrasinusoidal and interstitial, sometimes nodular. Mostly mild fibrosis	Mostly intrasinusoidal with a minor interstitial component, rarely nodular. Mostly mild fibrosis	Interstitial infiltration with prominent reticulin fibrosis
Immunophenotype				
CD11c	+/− (low intensity)	++ (moderate intensity)	++ (high intensity)	++ (high intensity)
CD25	−	−−	−−	++
CD103	−−	−/+	++	++
CD123	−−	−/+	−/+ (dim)	++ (bright)
CD200	++	−/+	−	++
DBA.44	+/−	+	+/−	++
Annexin A1	−−	−−	−−	++
Cyclin D1	−−	−−	−−	++ (weak and variable expression)
Cyclin D3	−−	+/−	NA	−
Molecular diagnostics	Most frequently mutations in KLF2, NOTCH, and NF-kappaB pathway genes. *MYD88* mutations in approx. 8%, one-third of which are L265P	Most frequently mutations in *BCOR*, *CCND3*, and NOTCH pathway genes. *MAP2K1* mutations are rare (approx. 10%). Only very rare reports of *MYD88* L265P and *BRAF* V600E mutations	*MAP2K1* mutations in approx. 30%. *BRAF* wildtype	*BRAF* V600E mutations in 90–100%

Abbreviations: *HCL*, hairy cell leukemia; *NA*, not available; *SBLPN*, splenic B-cell lymphoma with prominent nucleoli; *SDRPL*, splenic diffuse red pulp lymphoma; *SMZL*, splenic marginal zone lymphoma

Immunophenotype: −− less than 10% of cases positive; − 10–25%; −/+ 25–50%; +/− 50–75%; + 75–90%; ++ 90–100%

### Splenic diffuse red pulp small B-cell lymphoma

SDRPL was first recognized as a provisional entity separate from SMZL in the 2008 WHO classification. In the 2017 revised 4th edition, it was grouped together with HCLv in the category of splenic B-cell lymphoma/leukemia, unclassifiable. While the ICC [2] maintains the same classification scheme as the WHO revised 4th edition, in the WHO 5th edition, it is now separately classified. As its name implies, SDRPL shows extensive infiltration of red pulp cords and sinusoids with effacement of the white pulp [40–44]. In the bone marrow, SDRPL is typically characterized by an intrasinusoidal pattern of infiltration, but interstitial and nodular infiltrates are usually also present. Staining for DBA.44 is positive in the large majority of cases [40, 41, 45] and CD103 is expressed in approximately one-third of cases [43]. Expression of CD11c is positive in the large majority of cases with a fluorescent intensity by flow cytometry that is higher than SMZL but lower than HCL [46]. Expression of cyclin D1 and Annexin A1 has only very rarely been reported in isolated cases [40, 41, 47]. Immunohistochemistry for cyclin D3 is a promising marker as it is reported to be expressed in approximately 70% of SDRPLs and not in other splenic B-cell lymphomas, but the current data are still limited [48, 49].

Sequencing studies of SDRPL have shown recurrent mutations in *BCOR* (in 24%), *CCND3* (in 21–26%), genes involved in the NOTCH pathway (*NOTCH1*, *NOTCH2*, *SPEN* (in 17%)), and *MAP2K1* (in 7–10%) [48, 50–52]. *MYD88* L265P mutation and *BRAF* V600E mutations have been reported only very rarely [50, 52].

Four cases of SDRPL were submitted to the workshop. The case by Miekan Stonhill (LYWS-1140) illustrated the difficulty in establishing the diagnosis without a splenectomy specimen. This case carried a *BCL2* rearrangement which is only rarely found in indolent B-cell lymphoma outside the context of follicular lymphoma and had not previously been reported in SDRPL [53]. In a case submitted by Lucile Baseggio, a typical *BCOR* mutation was detected (LYWS-1397). Tapan Mahendra Bhavsar (LYWS-1150) (Fig. 1c and 1d) and Juan F. Garcia (LYWS-1211) submitted two additional cases of SDRPL diagnosed on splenectomy.

### Splenic B-cell lymphoma/leukemia with prominent nucleoli/hairy cell leukemia variant

Splenic B-cell lymphoma/leukemia with prominent nucleoli (SBLPN) is a new entity in the 5th edition of the WHO classification, which contains HCLv and also cases that were previously classified as CD5 negative B-cell prolymphocytic leukemia (B-PLL) [1]. The term HCLv was removed in the 5th edition of the WHO classification as these cases are thought to be biologically unrelated to HCL. The ICC [2] continues to use the term HCLv with B-PLL retained as a separate entity. Considering this recent change in classification, most of the features of SBLPN/HCLv described below are based on studies on the entity of HCLv. Patients present with splenomegaly with anemia and thrombocytopenia in a subset of patients but without the monocytopenia seen in HCL [54] and white blood cell counts are often higher than in HCL. Hepatomegaly is found in 20% of patients, and peripheral lymphadenopathy is rare. The neoplastic cells are characterized by prominent nucleoli and fine cytoplasmic projections resembling those of hairy cell leukemia (HCL). The pattern of infiltration in the spleen is similar to that of SDRPL and HCL with predominant infiltration of the red pulp. In the bone marrow, the pattern of involvement is typically intrasinusoidal with a minor interstitial component while nodular infiltrates occur only very rarely [55]. The interstitial infiltration with diffuse reticulin fibrosis which is typical of HCL is not a feature of SBLPN/HCLv.

Immunophenotypically SBLPN/HCLv expresses CD11c and often CD103, whereas CD123, CD25, annexin A1, and cyclin D1 are negative [56–58].

Molecular data on SBLPN/HCLv are limited. Recurrent mutations have been reported in *MAP2K1*, *TP53*, *U2AF1*, *KMD6A*, *CREBBP*, and *ARID1A*. *MAP2K1* mutations have been found in about 30% of SBLPN/HCLv [57, 59–62] and since they are only rarely found in SMZL and SDRPL, they can help in the differential diagnosis [24, 50]. *TP53* mutations are detected in approximately one-third of SBLPN/HCLv and are associated with a worse prognosis [63].

Distinguishing between SBLPN/HCLv and SDRPL can be difficult as both show a similar pattern in the spleen and the bone marrow. Helpful features are the presence of prominent nucleoli in SBLPN/HCLv while the chromatin in SDRPL is more condensed. If present, plasmacytic differentiation favors SDRPL. The immunophenotypes of these two entities are largely overlapping but CD103 expression is more common in SBLPN/HCLv and Cyclin D3 expression favors SDRPL but this staining is currently not widely available.

While evaluation of the peripheral blood and bone marrow can sometimes be diagnostic, it can be difficult or impossible to differentiate between the different types of splenic small B-cell lymphomas in the absence of splenic tissue for evaluation. In this situation, a diagnosis of splenic B-cell lymphoma, unclassifiable can be made [64, 65].

## Diagnosing transformation in marginal zone lymphoma

**Fig. 2:**
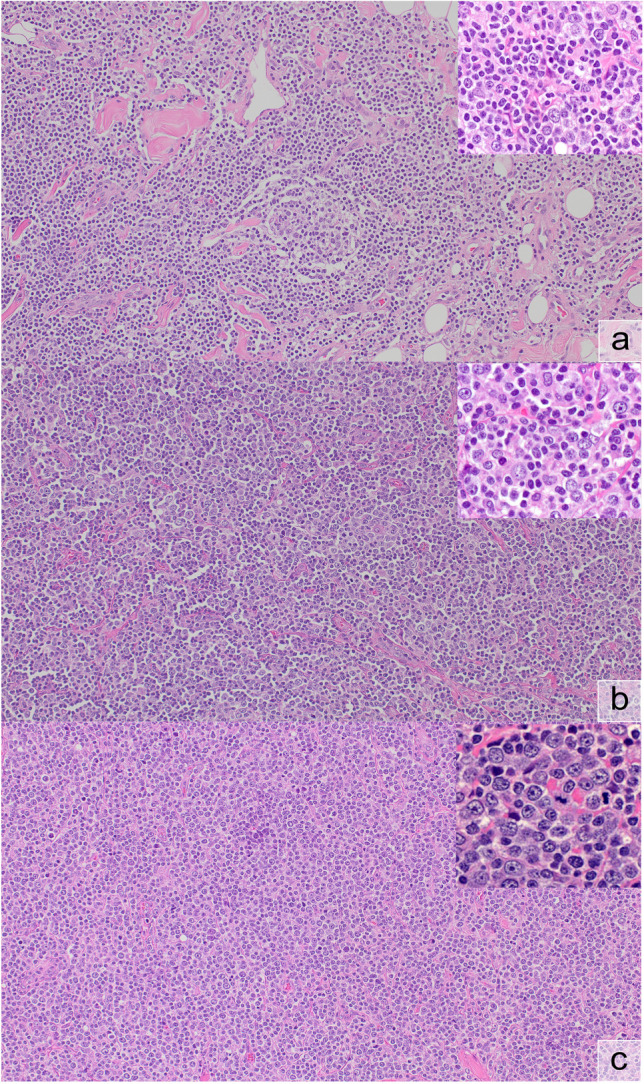
Transformation of nodal marginal zone lymphoma (NMZL). In this case (LYWS-1273), some areas **a** still showed conventional NMZL with low-grade cytology with few admixed large cells (inset), some areas **b** showed an increase in large cells (inset), and some areas **c** showed confluent aggregates of large cells compatible with transformation (inset); the few admixed small cells were almost exclusively T-cells

A diagnosis of transformation of MZL to diffuse large B-cell lymphoma carries important clinical consequences with respect to treatment and prognosis. Although transformation can usually be diagnosed or excluded with confidence, some cases show borderline features where the definition becomes more subjective. Transformation in MZL to DLBCL is defined as the presence of confluent sheets of blasts (in SMZL effacing the architecture with loss of red/white pulp demarcation) but since MZL can show a cytological spectrum with smaller and larger cells, it can be difficult to distinguish true blasts from larger “non-blastic” MZL cells including monocytoid cells. Also, a “sheet of blasts” has not been clearly defined. It has been proposed to diagnose transformation in the presence of clusters of at least 20 large cells [66] or if the large cells comprise more than 20% of the neoplastic population [67] but at present, there is no consensus. Case LYWS-1273 (April Chiu) showed some areas of low-grade lymphoma, some area with increased large cells but not sufficient for a diagnosis of transformation. In addition, there were some areas containing sheets of large neoplastic cells assessed as unequivocal transformation (Fig. 2). The difficulty in making a definitive diagnosis of transformation in some cases was discussed during the workshop in both SMZL (LYWS-1268, Pascale Cervera) as well as NMZL (LYWS-1316, Cara Monroe; LYWS-1465, Jan Bosch-Schips).

Transformation of SMZL to DLBCL was illustrated by cases LYWS-1115 by Kenneth Ofori, LYWS-1264 by Pascale Cervera, and LYWS-1332 by Marta Grau. In LYWS-1115, the SMZL component showed a complex karyotype which has been reported in association with an increased risk of transformation [68]. In LYWS-1332, both the SMZL and the DLBCL contained *TNFAIP3* mutations with an additional *MYD88* L265P mutation found only in the transformed component. This is consistent with the findings reported in abstract form in this meeting by the same group in which mutations in *TNFAIP3* and *TP53*, loss of 9p21.3, and gains of 6p were found to be acquired during SMZL transformation [69].

In cases of NMZL transformation to DLBCL submitted to the workshop, *TP53* mutations were also found in 2 cases (LYWS-1136, Natalia Papaleo; LYWS-1273, April Chiu) and *TNFAIP3* mutations were found in 2 cases (LYWS-1465; LYWS-1273). Other findings in NMZL with transformation to DLBCL were the association with a *NOTCH3* mutation (LYWS-1090, Julie Y. Li) and EBV positivity (LYWS-1136). A case submitted by Ahu Senem Demiröz (LYWS-1281) found classic Hodgkin lymphoma (cHL) adjacent to NMZL, but the clonal relationship between these two components could not be investigated due to limited material. Transformation of indolent B-cell lymphoma to cHL is a rare but well-known phenomenon in CLL. It has also been reported in MZL as single case studies. Some of these confirmed a clonal relationship between the MZL and cHL [68, 70–74].

Very rarely progression in SMZL consists of an increase in prolymphocytes which can be accompanied by expression of cyclin D1 in absence of a *CCND1* rearrangement as illustrated by case LYWS-1295 submitted by Xiaohui Zhang [72, 75].

## T follicular helper cell hyperplasia in marginal zone lymphoma

**Fig. 3:**
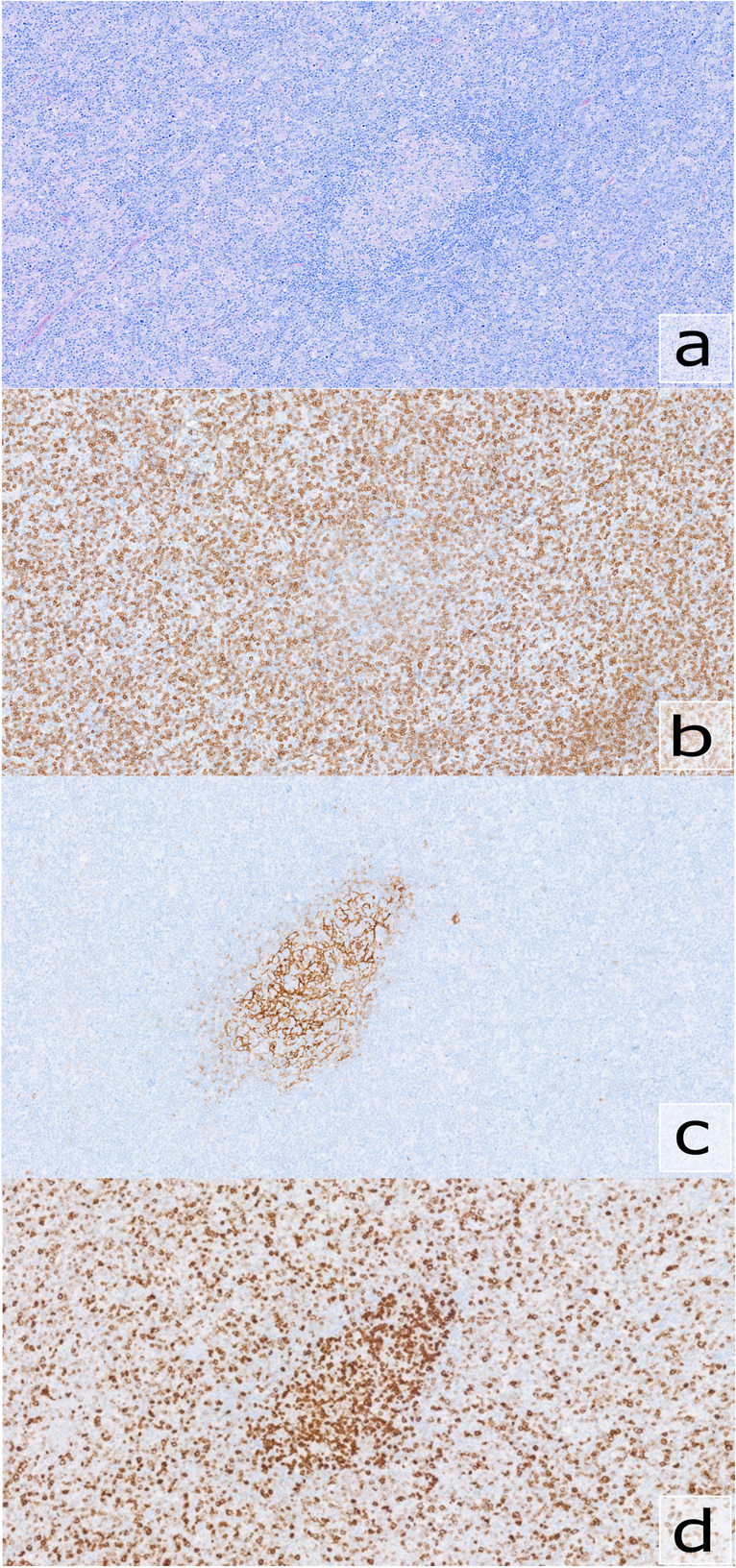
Marginal zone lymphoma with increased T follicular helper (TFH) cells. In this case (LYWS-1051), a follicular and diffuse pattern of increased TFH cells was seen (**a**) with numerous CD5-positive T-cells (**b**) within a germinal center remnant (CD23, **c**). The T-cells show expression of ICOS (**d**), simulating a T-cell lymphoma. Clonality studies showed a B-cell clone as well as mutations associated more frequently with B-cell lymphomas and absence of clonality of T-cells as well as absence of typical mutations associated with TFH derived lymphomas

Low-grade B-cell lymphomas, including MZLs, are accompanied by a rich tumor micro-environment that plays a critical role in cell survival and proliferation. T-cells form an important part of this milieu and in MZLs, it is possible to demonstrate the presence of a large reactive T-cell component. In some cases, the presence of T follicular helper (TFH) cells can be so extensive that a differential diagnosis between MZL with extensive TFH cells and a T-cell lymphoma (most commonly a nodal TFH cell lymphoma — nTFHL) with an associated B-cell component might be considered. Although increased TFH cells in MZL have been recognized in previous works [76, 77], this phenomenon was investigated more thoroughly in a paper from 2019 by Egan et al. [78] where the authors identified an increase in PD1+ cells in two-thirds of NMZLs. The PD1+ cells were mostly present in a follicular pattern at the periphery and/or the center of follicles but in a small number of cases, a diffuse pattern of increased PD1+ cells could be seen. Hurwitz and colleagues shortly thereafter reported on three additional cases of MZL with increased TFH cells, which were at some point under consideration as a T-cell lymphoma [79]. Reflecting this increased recognition of TFH expansion in MZL, multiple cases submitted to the workshop dealt with this diagnostic pitfall. Most of these were NMZLs (*n*=8) (summarized in Table 2), but TFH increases were also found in pediatric MZL (LYWS-1399, Gioia Di Stefano) and SMZL (LYWS-1151, Marie Parrens). There was a strong female predominance present among the submitted cases (7/8 of nodal cases were female), a feature also present, although less pronounced, in the previously published series [78, 79] (30/48 and 2/3 female cases, respectively). The pattern of TFH expansion in NMZL was nodular in most of the cases but a diffuse pattern was observed in two cases (LYWS-1051, Leonie Frauenfeld; LYWS-1168, Atif Saleem) (Fig. 3). Rebecca King submitted a case (LYWS-1082) of NMZL with a very extensive proliferation of TFH cells to the extent that the underlying B-cell lymphomas was not recognizable in some of the tissue blocks that were taken from the involved lymph node. To further complicate the differential diagnosis with nTFHL, some cases showed scattered EBER-positive cells (LYWS-1168; LYWS-1347, Udit Kamlesh Naik; LYWS-1433, Shunyou Gong).

**Table 2:** Description of nodal marginal zone lymphoma cases with increased T follicular helper cells

Case number	Age	Sex	Disease localization	Clonality	NGS	Cytogenetics/FISH
LYWS-1031	60	M	Multiple	B+, T−	*NOTCH2*, *CREBBP*, and *KLF2 *mutations	*MYC* BA negative
LYWS-1037	53	F	Axillary	B+, T−	Negative for *TET2*, *RHOA*, *DNMT3A*, and *IDH2* mutations	n.a.
LYWS-1039	77	F	Multiple	B+, T−	*NOTCH2* and *KLF2* mutations	Abnormal karyotype FISH negative for *IGH* and *TCL1A* rearrangements
LYWS-1051	60	F	Axillary, inguinal, kidney	B+, T−	*CD70*, *IRF4*, *TMSB4X*, and *BTG2 *mutations	n.a.
LYWS-1082	78	F	Inguinal, pelvic	B+, T−	n.a.	n.a.
LYWS-1168	91	F	Mediastinal and hilar	B+, T−	*NOTCH2*, *CCND3*, *IRF8*, and *NOTCH1 *mutations	n.a.
LYWS-1347	55	F	Multiple	B+, T−	*SPEN* (2x) and *TNFAIP3* mutations	Negative for t(11;14) t(14;18) *BCL6* :: *MALT1* rearrangement
LYWS-1433	57	F	Inguinal	B+, T−	Negative for mutations in *MYD88*

Clonality studies for immunoglobulin (IG) and T-cell receptor genes (TR) are essential to distinguish between B- and T-cell lymphoma in these cases. Clonality studies for IG and TR were performed in all cases and each of the cases showed a B-cell clone. T-cell clonality studies were polyclonal except for one case of SMZL (LYWS-1151) where mutations more commonly associated with T-cell lymphomas were identified (in *FAS* and *KMT2C*). The clinical course in this case did not support a diagnosis of T-cell lymphoma given disease regression after splenectomy.

Sequencing analysis results for genes involved in B-cell lymphoma were described in 6 cases. Recurrent mutations were only found for *NOTCH2* (in three cases; LYWS-1031 and LYWS-1039, Stephanie N. Hurwitz; LYWS-1151) and *KLF2* (in two cases; LYWS-1031 and LYWS-1039). Absence of mutations in genes associated with nTFHL (i.e., *RHOA*, *IDH2*, *DNMT3A*, *TET2*) can help to further exclude nTFHL, especially in cases with a diffuse pattern of TFH-cell hyperplasia (Fig. 3). No mutations in these genes were identified in the 5 cases investigated (LYWS-1037, Jennifer R. Chapman; LYWS-1031; LYWS-1039; LYWS-1051; LYWS-1151).

## Pediatric nodal marginal zone lymphoma and related entities

Pediatric nodal marginal zone lymphoma (PNMZL) is a rare, indolent B-cell lymphoma arising in children and young adults [80, 81] although cases with similar features have been reported in middle-aged adults [82]. It shows a strong male predominance, preference for head and neck lymph nodes or the Waldeyer’s ring, and very indolent behavior as well as low stage at presentation [80]. Its distinction from “conventional” NMZL is important as most patients are cured by simple excision with or without local irradiation. The typical morphological features include expanded marginal zones with partial preservation of the follicular architecture and follicular changes similar to progressively transformed germinal centers (PTGC) including germinal center fragmentation and regression with expansion of the mantle zone. Sometimes an interfollicular or diffuse component is present, which is more reminiscent of “conventional” NMZL. The extrafollicular microenvironment might include increased plasma cells and eosinophils. The immunophenotype does not differ from that of “conventional” NMZL, notably lacking specific markers and showing aberrant expression of CD43 in a proportion of cases. Light chain restriction of the secretory differentiated component might also be present. Rare cases might variably express CD10 posing diagnostic problems in differentiating from a follicular lymphoma [83]. Increased PD1-positive intrafollicular TFH-cells are commonly noted, a feature that might help with differentiating PNMZL from pediatric-type follicular lymphoma (PTFL) [83]. Cytogenetic features include trisomy 18 and more rarely trisomy 3 [84]. Detection of B-cell clonality is almost indispensable for the diagnosis as in children atypical marginal zone hyperplasia in the tonsils [85] or in the lymph nodes [86] might simulate PNMZL (see further discussion later). A whole exome approach identified only a few mutations (none recurrent) in 6 cases of PNMZL including one in *AMOTL1*, a gene recurrently mutated in SMZL [87] Recently genetic abnormalities overlapping with those of PTFL have been detected in the majority of PNMZL [88] or in cases displaying intermediate/mixed morphological features between PNMZL and PTFL [89] supporting a similar pathogenesis in the two lymphomas with some authors advocating the merging of the two entities into one single disease [88, 90]. It is noteworthy that about one-third of PNMZL show no genetic lesions detectable by current technology.

**Fig. 4:**
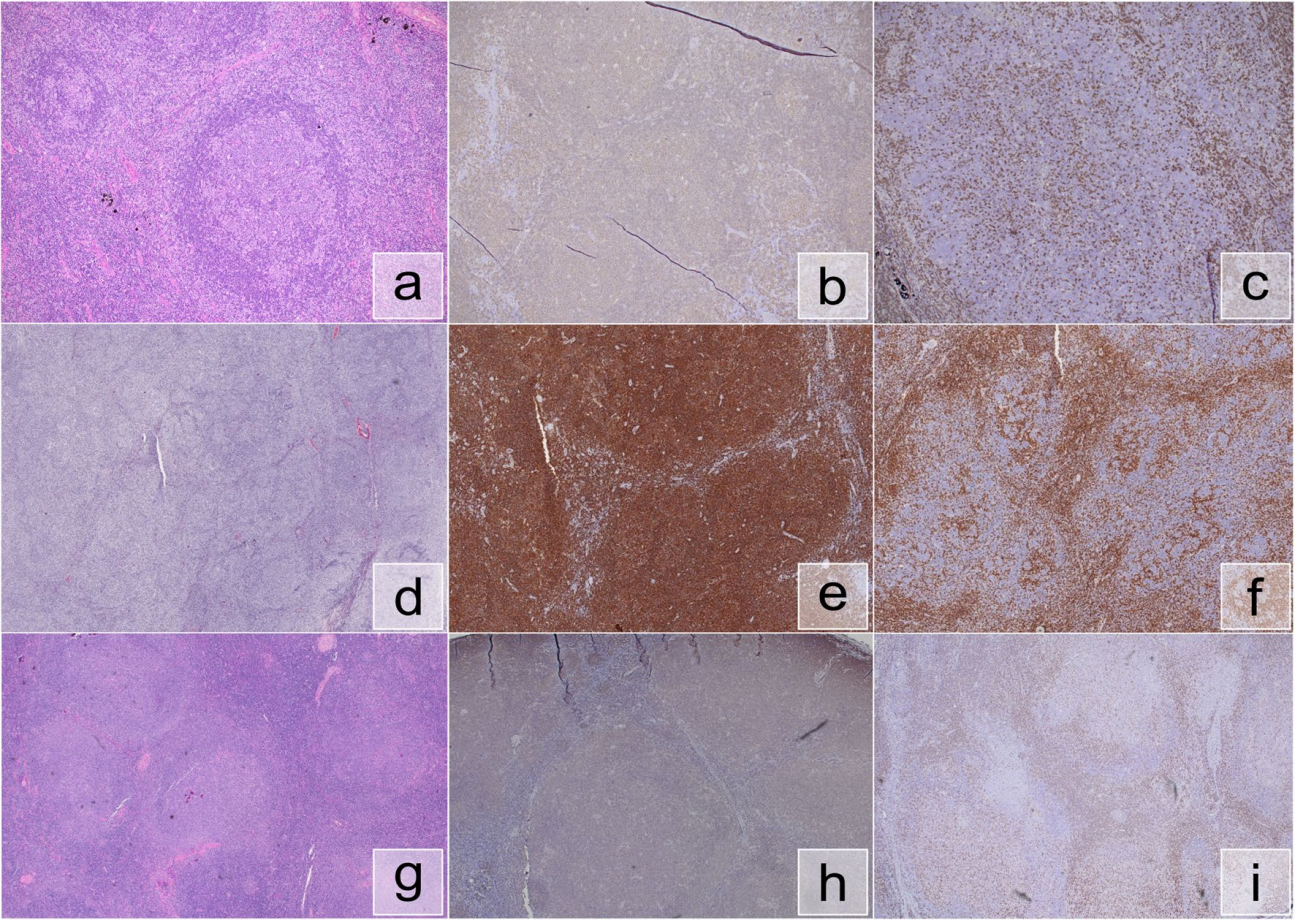
Pediatric nodal marginal zone lymphoma (PNMZL) and pediatric marginal zone hyperplasia (PMZH). **a** LYWS-1311 represents a typical PNMZL with PTGC-like changes and expanded marginal zones; **b** same case, CD20; **c** same case, CD5; **d** LYWS-1353 showed overlapping features between PNMZL and PTFL, including coalescing germinal centers; **e** same case, CD20; **f** same case, CD3; **g** LYWS-1160, PNMZH showed features of a PNMZL but was polyclonal, showed no mutations, and was associated with *H. influenzae*; **h** same case, CD20; **i** same case, CD5

Seven cases of PNMZL were submitted in this workshop (Table 3) among which four were young adults (age 18–33 years) and three teenagers (age 13–16 years). The well-known male predominance was confirmed in our series (M:F=6:1) but the topography of the lesions was not completely typical with only two cases in the head and neck region; the remaining cases were in the upper arm (2), axilla (1), inguinal region (1), and the tonsil (1). B-cell clonality was detected in all cases while T-cell clonality was tested in only 3 cases and was negative in all. The morphology showed some variability with some very typical cases showing well defined marginal zone expansion and some PTGC-like changes (for instance, case LYWS-1311, Ioannis Anagnostopoulos, Fig. 4a) while others showed some diffuse interfollicular growth reminiscent of “conventional” NMZL (e.g., case LYWS-1301, Elaine Jaffe). Some cases showed a “hybrid” morphology, intermediate between a PNMZL and a PTFL (e.g., LYWS-1353, Zheng Cao and LYWS-1399 Gioia Di Stefano — Fig. 4d). Cytogenetics was performed in one case (LYWS-1294, Wen-Hsuan Wendy Lin) which showed a tetraploid karyotype with additional losses of chromosomes 1, 3, 4, 5, and 7. The same case was also negative for BCL6 rearrangement by FISH using break-apart probes. FISH was also performed in case LYWS-1353 which was negative for *BCL2*, *BCL6*, and *MYC* rearrangements. Advanced molecular studies were performed in cases LYWS-1177, LYWS-1301, and LYWS-1311. Case LYWS-1177 (Catherine Chassagne-Clement) showed no alterations by whole transcriptome analysis but this technique is notably not able to detect mutated targets which are expressed at low levels. Case LYWS-1301 was tested using an Illumina TruSight Oncology Panel v2, and showed mutations in *MAP2K1* and *TNFRSF14*. Case LYWS-1311 was tested by a custom designed panel containing 42 genes commonly mutated in B-cell malignancies (including *TNFRSF14*, *MAP2K1*, and *IRF8*) but no mutations were detected.

**Table 3:** Description of pediatric nodal marginal zone lymphoma cases

Case number	Age	Sex	Disease localization	Clonality	NGS	Cytogenetics/FISH
LYWS-1177	16	F	*Tonsil*	B+, T−	Negative by whole transcriptome	n.a.
LYWS-1294	13	M	*Inguinal*	B+, T−	n.a.	Tetraploid, loss of chromosomes 1, 3, 4, 5, and 7. *BCL6* break apart FISH neg.
LYWS-1301	16	M	*Upper arm*	B+	*MAP2K1* (VAF 7.9%) and *TNFRSF14* (VAF 8.0%) mutations	n.a.
LYWS-1308	19	M	*Submental*	B+	n.a.	n.a.
LYWS-1311	24	M	*Upper arm*	B+	Negative (custom 42-gene panel)	n.a.
LYWS-1353	18	M	*Cervical*	B+	n.a.	*MYC*, *BCL2*, *BCL6* neg.
LYWS-1399	33	M	*Axillary*	B+, T oligo/poly	n.a.	n.a.

Case LYWS-1160 (Alberto Zamò) showed one of the main differential diagnostic problems in NMZL. The 8-year-old patient presented with enlarged cervical lymph nodes, measuring up to 5 cm. The histological picture showed a marked expansion of cortical follicles with prominent marginal zones. The germinal centers were partly preserved but sometimes showed fragmentation combined with mantle zone expansion reminiscent of PTGC (Fig. 4g). The immunophenotype was not conclusive but an increased proliferation of the marginal zone areas was noted in the Ki-67 staining. Light chain restriction was not present and clonality studies were also negative. A 42-gene panel for mutations commonly present in B-cell lymphoma yielded negative results. During the diagnostic work-up, microbiological tests from fresh tissue provided a positive result for *Haemophilus influenzae* and a final diagnosis of pediatric nodal marginal zone hyperplasia (*H. influenzae* related) was made. Pediatric nodal marginal zone hyperplasia is a reactive process arising in children with no sex predilection showing a median age younger than PNMZL (12 years) and is strongly related to the presence of *H. influenzae* in the lymphoid tissues (as demonstrable either by culture or by molecular studies). Its histologic appearance is deceptively similar to PNMZL and skewed light chain expression (most often lambda) can be demonstrated in many cases but molecular clonality studies are negative. Besides the distinction from PNMZL, because of the presence of progressively transformed germinal centers, the differential diagnostic spectrum also includes nodular lymphocyte predominant Hodgkin lymphoma/nodular lymphocyte predominant B-cell lymphoma; Table 4 shows the main diagnostic features of these three entities. The process is very similar to atypical marginal zone hyperplasia of the tonsil or appendix [85] where a predominant lambda light chain restriction is also noted. A similar histopathological picture has been reported in activated phosphoinositide 3-kinase δ syndrome (APDS), in which increased monocytoid B-cells and focal positivity for EBV and CMV have been described [91]. Expression of CD43 in B-cells has been sometimes reported in pediatric nodal marginal zone hyperplasia, another feature similar to PNMZL.

**Table 4:** Differential diagnostic criteria for pediatric (atypical) marginal zone hyperplasia, pediatric nodal marginal zone lymphoma, and nodular lymphocyte predominant Hodgkin lymphoma

	Atypical MZH	PNMZL	NLPHL
Marginal zone hyperplasia	+	+	−/+
Follicular hyperplasia	+/−	+/−	−
PTGC-like changes	+	+	+
Light chain restriction	+/− (lambda)	+/−	Rare
Ki-67	High in GC	Relatively increased in MZ and high in residual GC	High in residual GC and LP cells
TFH-rosettes	Absent	Absent (increased TFH lymphocytes)	Present
LP-cells	Absent	Absent (sometimes cytologic atypia)	Present
Immunoglobulin clonality analysis	Polyclonal	Clonal	Usually polyclonal (due to low tumor cell content)
Mutational analysis	Negative	Sometimes *TNFRSF14*, *MAP2K1*, *IRF8* mutations	Usually neg. (low tumor cell content) or *STAT6*, *SOCS1*, etc.

*MZH*, marginal zone hyperplasia; *PNMZL*, pediatric nodal marginal zone lymphoma; *NLPHL*, nodular lymphocyte-predominant Hodgkin lymphoma; *ISH*, in situ hybridization; *GC*, germinal centers; *MZ*, marginal zone; *LP*, lymphocyte-predominant; *TFH*, T follicular helper

## Conclusion

The diagnostic approach to marginal zone lymphoma, be it nodal, splenic, or extranodal, is shifting from a diagnosis of exclusion to one defined by specific criteria; however, diagnostic difficulties still exist as a result of a lack of a specific immunophenotype and subtle morphological features. In this workshop, we analyzed and discussed several issues related to the diagnosis of splenic and nodal marginal zone lymphomas, potential pitfalls, and the criteria for transformation. The most important conclusions are summarized in Box 1.

**Table:** 

Box 1 • Distinction between SMZL, SDRPL, and SBLPN/HCLv requires integration of clinical features, immunophenotype, and morphology in blood, bone marrow, and spleen. Next generation sequencing can be of added value. If the spleen is not available for evaluation, distinction may not be possible. • TFH cells can be increased in all MZLs, which should be distinguished from T-cell lymphoma by performing appropriate immunohistochemical and molecular studies. • Strict criteria for transformation in MZL are lacking and borderline cases suspicious for transformation do occur. Risk of transformation may be associated with genetic abnormalities (*NOTCH3* mutations, complex karyotype). • PNMZL is not limited to the pediatric age group and can occur in young adults. • PNMZL might have overlapping features with PTFL but the possible relationship remains to be determined. • B-cell clonality detection is vital to differentiate PNMZL from reactive conditions such as atypical marginal zone hyperplasia.	

### Supplementary information

ESM 1 (DOCX 24 kb)

## Data Availability

Not applicable
