# Correction to: The many faces of nodal and splenic marginal zone lymphomas. A report of the 2022 EA4HP/SH lymphoma workshop

**DOI:** 10.1007/s00428-023-03646-y

**Published:** 2023-09-11

**Authors:** Alberto Zamò, Michiel van den Brand, Fina Climent, Laurence de Leval, Stefan Dirnhofer, Lorenzo Leoncini, Siok‑Bian Ng, Sarah L. Ondrejka, Leticia Quintanilla‑Martinez, Lorinda Soma, Andrew Wotherspoon

**Affiliations:** 1 Institute of Pathology, University of Würzburg, Josef-Schneider-Str. 2, 97080 Würzburg, Germany; 2 Pathology-DNA, Location Rijnstate Hospital, Wagnerlaan 55, 6815AD Arnhem, The Netherlands; 3 Department of Pathology, Radboud University Medical Center, Nijmegen, The Netherlands; 4 Department of Pathology, Hospital Universitari de Bellvitge-IDIBELL, L’Hospitalet de Llobregat, Barcelona, Spain; 5 Department of Laboratory Medicine and Pathology, Institute of Pathology, Lausanne University Hospital and Lausanne University, Lausanne, Switzerland; 6 Institute of Medical Genetics and Pathology, University Hospital Basel, University of Basel, Basel, Switzerland; 7 Department of Medical Biotechnology, Section of Pathology, University of Siena, Siena, Italy; 8 Department of Pathology, Yong Loo Lin School of Medicine, National University of Singapore, Singapore, Singapore; 9 Cancer Science Institute of Singapore, National University of Singapore, Singapore, Singapore; 10 Pathology and Laboratory Medicine Institute, Cleveland Clinic, Cleveland, OH USA; 11 Institute of Pathology and Neuropathology, Eberhard Karls University of Tübingen and Comprehensive Cancer Center, University Hospital Tübingen, Tübingen, Germany; 12 Department of Pathology, City of Hope Medical Center, Duarte, CA USA; 13 Department of Histopathology, Royal Marsden Hospital, London, UK

**Correction to** **:** **Virchows Archiv**

**https://doi.org/10.1007/s00428-023-03633-3**

The article title of the published version of the above article was incomplete. The correct title is shown as follows:

**“The many faces of nodal and splenic marginal zone lymphomas. A report of the 2022 EA4HP/SH lymphoma workshop”**

The original article has been corrected.

