# Supplementary material for: The many faces of nodal and splenic marginal zone lymphomas. A report of the 2022 EA4HP/SH lymphoma workshop
Source: Virchows Arch. 2023 Sep 1;483(3):317–31. doi: 10.1007/s00428-023-03633-3 (PMC10542713; doi:10.1007/s00428-023-03633-3)
Supplement: Supplementary file 1 — (DOCX 24 kb) [file 428_2023_3633_MOESM1_ESM.docx]

| **Supplementary Table 1 – Summary of all 42 cases submitted to the workshop session** | | | | | | | | | | | | |
| --- | --- | --- | --- | --- | --- | --- | --- | --- | --- | --- | --- | --- |
| ***Splenic marginal zone lymphoma and its differential diagnosis*** | | | | | | | | | | | | |
| *Case number* | *Submitter* | *Age* | | | | *Sex* | *Disease localization* | *Clonality* | *NGS* | *Cytogenetics / FISH* | *Panel diagnosis* | *Feature/ comment* |
| LYWS-1040 | Miekan Stonhill, Boston, USA | 61 | | | | F | Spleen, bone marrow | n.a. | *CXCR4* mutation | *IGH::BCL2* translocation | SDRPL |  |
| LYWS-1052 | Sohaib Al-Khatib, Irbid, Jordan | 29 | | | | M | Spleen | n.a | n.a. | n.a. | SMZL | Presentation with recurrent lymphocytopenia and fever |
| LYWS-1150 | Tapan Bhavsar, Washington, USA | 70 | | | | F | Spleen | n.a. | n.a. | n.a. | SDRPL |  |
| LYWS-1151 | Marie Parrens, Bordeaux, France | 53 | | | | M | Spleen | B+, T+ | Mutations in *NOTCH2*, *TNFRSF14*, *FAS* and *KMT2C* | Negative for *BCL2*/ *BCL6* rearrangements or del1p36 | SMZL | Associated clonal TFH population |
| LYWS-1165 | Gabriel Caponetti, Philadelphia, USA | 79 | | | | F | Spleen, lymph nodes, bone marrow | n.a. | Mutations in *BIRC3* and *TP53* | Normal karyotype. No rearrangements of *BCL6*, IGH or *MALT1* | SMZL | *BIRC3* mutations |
| LYWS-1211 | Juan Garcia, Madrid, Spain | 71 | | | | M | Spleen, bone marrow | n.a. | Negative for *BRAF* mutation | Negative for del7q, del17p and *BCL2*/ *CCND1* rearrangements | SDRPL | Overlap with HCL-v |
| LYWS-1295 | Xiaohui Zhang, Tampa, USA | 71 | | | | M | Spleen, bone marrow | n.a. | Mutations in *NOTCH2* and *TP53* | Complex karyotype. FISH positive for del17p and negative for rearrangements of *CCND1*, *BCL2*, *BCL6*, *IGH*, *MYC* and *MALT1* | SMZL | Increased prolymphocytes and expression of cyclin D1 |
| LYWS-1397 | Lucille Baseggio, Lyon, France | 79 | | | | M | Spleen | n.a. | *BCOR* mutation. No mutations in *BRAF* or *NOTCH2* | Trisomy 12 and trisomy 5. | SDRPL | Overlap with SMZL and HCL-v |
| ***Diagnosing transformation in marginal zone lymphoma*** | | | | | | | | | | | | |
| *Case number* | *Submitter* | | | | *Age* | *Sex* | *Disease localisation* | *Clonality* | *NGS* | *Cytogenetics / FISH* | *Panel diagnosis* | *Feature/ comment* |
| LYWS-1090 | Julie Li, Boston, USA | | | | 75 | M | Multiple | B+ | *NOTCH3* mutation | No rearrangements of *BCL6*, *MALT1*, *CCND1*, *CCND2* or *CCND3*. Gain of *MALT1* | Transformed NMZL | *NOTCH3* mutation |
| LYWS-1115 | Kenneth Ofori, New York, USA | | | | 71 | M | Spleen | B+ | n.a. | Complex karyotype | Transformed SMZL | Complex karyotype in SMZL |
| LYWS-1136 | Natalia Papaleo, Barcelona, Spain | | | | 89 | M | Multiple | n.a. | No *MYD88* mutation | *BCL6* rearrangement. No rearrangement of *BCL2*/ *MYC* | Transformed NMZL | EBV-associated |
| LYWS-1264 | Pascale Cervera, Paris, France | | | | 59 | F | Spleen | n.a. | n.a. | n.a. | Transformed SMZL | Large cells in hilar lymph nodes |
| LYWS-1268 | Pascale Cervera, Paris, France | | | | 53 | M | Spleen | n.a. | n.a. | n.a. | SMZL | Aggressive behavior |
| LYWS-1273 | April Chiu, Rochester, USA | | | | 72 | M | Occipital lymph node | n.a. | Likely pathogenic mutations in *KMT2D*, *TNFAIP3*, *TP53*, *B2M*, *FAS* and *TET2*. VUS in *KMT2D*, *MYD88*, *EP300*, *DDX3X*, *BTG1* and *TET2* | No rearrangements of *BCL2*, *BCL6* or *MYC* | Transformed NMZL |  |
| LYWS-1316 | Cara Monroe, Los Angeles, USA | | | | 51 | F | Multiple | B+, T+ | *CREBBP* mutation, *CDKN2A* copy number loss | n.a. | Transformed NMZL | Associated peripheral T-cell lymphoma |
| LYWS-1332 | Marta Grau, Barcelona, Spain | | | | 65 | F | Multiple | B+ | Mutations in *SPEN*, *KMT2D*, *NOTCH2*, *TNFAIP3*, *KLF2*, *BCL10*, *BRAF*, *MYD88*, *CXCR4* | No BCL6 rearrangement, 3 copies of MYC | Transformed NMZL | Plasmacytic differentiation, acquired *MYD88* mutation |
| LYWS-1465 | Jan Bosch-Schips, Barcelona, Spain | | | | 58 | F | Multiple | B+ | Mutations in *KMT2D*, *TNFAIP3*, *CD58*, *SOCS1* and *MYC* | No rearrangements of *BCL2*, *BCL6* or *MYC* | Transformed NMZL |  |
| ***T follicular helper cell hyperplasia in marginal zone lymphoma*** | | | | | | | | | | | | |
| *Case number* | *Submitter* | | | | *Age* | *Sex* | *Disease localization* | *Clonality* | *NGS* | *Cytogenetics / FISH* | *Panel diagnosis* | *Feature/ comment* |
| LYWS-1031 | Stephanie Hurwitz, Philadelphia, USA | | | | 60 | M | multiple | B+, T- | *NOTCH2*, *CREBBP*, and *KLF2* | No *MYC* rearrangement | NMZL | *NOTCH2*, *CREBBP*, and *KLF2* mutations, large cell transformation |
| LYWS-1037 | Jennifer Chapman, Miami, USA | | | | 53 | F | axillary | B+, T- | Negative for *TET2*, *RHOA*, *DNMT3A*, and *IDH2* mutations | n.a. | NMZL | Initial external diagnosis of AITL |
| LYWS-1039 | Stephanie Hurwitz, Philadelphia, USA | | | | 77 | F | multiple | B+, T- | Mutations in *NOTCH2* and *KLF2* | Abnormal karyotype  FISH negative for *IGH* and *TCL1A* rearrangements | NMZL |  |
| LYWS-1051 | Leonie Frauenfeld, Tübingen, Germany | | | | 60 | F | axillary, inguinal, kidney | B+, T- | Mutations in *CD70*, *IRF4*, *TMSB4X* and *BTG2* | n.a. | NMZL |  |
| LYWS-1082 | Rebecca King, Rochester, USA | | | | 78 | F | inguinal, pelvic | B+, T- | n.a. | n.a. | NMZL | Initially externally diagnosed as PTCL-TFH |
| LYWS-1168 | Atif Saleem, Palo Alto, USA | | | | 91 | F | mediastinal and hilar | B+, T- | Mutations in *NOTCH2*, *CCND3*, *IRF8*, and *NOTCH1* | n.a. | NMZL | Rare EBER+ cells, diffuse extrafollicular distribution of the TFH component |
| LYWS-1347 | Udit Naik, Houston, USA | | | | 55 | F | multiple | B+, T- | *SPEN* (2x) and *TNFAIP3* mutations | Negative for t(11;14) t(14;18)  *BCL6* :: *MALT1* rearrangement | NMZL | Previous diagnosis of subcutaneous panniculitis-like T-cell lymphoma |
| LYWS-1433 | Shunyou Gong, Chicago, USA | | | | *57* | F | inguinal | B+, T- | Negative for mutations in *MYD88* | n.a. | NMZL | Expanded FDC meshworks (AITL-like), architecture partially preserved |
| ***Pediatric nodal marginal zone lymphoma and related entities*** | | | | | | | | | | | | |
| *Case number* | *Submitter* | | | *Age* | | *Sex* | *Disease localisation* | *Clonality* | *NGS* | *Cytogenetics / FISH* | *Panel diagnosis* | *Feature/ comment* |
| LYWS-1160 | Alberto Zamò, Würzburg, Germany | | | 8 | | M | Cervical | B-, T- | Negative | n.a. | Marginal zone hyperplasia | H. influenzae detected |
| LYWS-1177 | Catherine Chassagne-Clement, Lyon, France | | | 16 | | F | Tonsil | B+, T- | Negative by whole transcriptome | n.a. | PNMZL | Increased TFH cells |
| LYWS-1294 | Wen-Hsuan Wendy Lin, New York, USA | | | 13 | | M | Inguinal | B+, T- | n.a. | tetraploid, loss of chromosomes 1, 3, 4, 5, and 7. *BCL6* break apart FISH neg. | PNMZL | Clonal chromosome abnormalities in a tetraploid karyotype |
| LYWS-1301 | Elaine Jaffe, Bethesda, USA | | | 16 | | M | Upper arm | B+ | *MAP2K1* (VAF 7.9%) and *TNFRSF14* (VAF 8.0%) | n.a. | PNMZL |  |
| LYWS-1308 | TH Lee, Los Angeles, USA | | | 19 | | M | Submental | B+ | n.a. | n.a. | PNMZL |  |
| LYWS-1311 | Ioannis Anagnostopoulos, Würzburg, Germany | | | 24 | | M | Upper arm | B+ | Negative | n.a. | PNMZL |  |
| LYWS-1353 | Zheng Cao, Beijingm China | | | 18 | | M | Cervical | B+ | n.a. | *MYC*, *BCL2*, *BCL6* neg. | PNMZL |  |
| LYWS-1399 | Gioia Di Stefano, Florence, Italy | | | 33 | | M | Axillary | B+, T oligo/poly | n.a. | n.a. | PNMZL |  |
| ***Other cases with variable features*** | | | | | | | | | | | | |
| *Case number* | *Submitter* | | *Age* | | | *Sex* | *Disease localisation* | *Clonality* | *NGS* | *Cytogenetics / FISH* | *Panel diagnosis* | *Feature/ comment* |
| LYWS-1054 | Joshua Menke, Stanford, USA | | 72 | | | F | Multiple | n.a. | Mutations in *NFKB2* and *EP300* | Del4q, no *MYC* rearrangement | MZL | *NFKB2* 3’ activating mutation |
| LYWS-1084 | Joseph Rohr, Omaha, USA | | 20 | | | M | Small bowel | B- | n.a. | Complex karyotype | IPSID | Unusual phenotype, multiple microbial infections |
| LYWS-1147 | Tapan Bhavsar, Washington, USA | | 77 | | | F | Multiple | n.a. | n.a. | Cytogenetics: normal karyotype  FISH analysis: normal CLL and myeloma panels | NMZL | CD23 expression |
| LYWS-1148 | Tapan Bhavsar, Washington, USA | | 75 | | | M | Multiple | n.a. | n.a. | n.a. | CLL |  |
| LYWS-1250 | Bobbie Pelham-Webb, New York, USA | | 74 | | | F | Multiple | B+, weak T+ with PCB |  | 46,XX,i(18)(q10), add(21)(p12)[8]/ 47,idem, +18[5]/ 46,XX[6] | NMZL | EBV positive, autoimmune disease, iatrogenic immune supression |
| LYWS-1253 | Dorottya Laczko, Philadelphia, USA | | 80 | | | M | Submandibular and cervical | n.a. | *EZH2*, *TBL1XR1*, *TNFRSF14* | Positive for *BCL2* rearrangement | Follicular lymphoma | Mimicking NMZL |
| LYWS-1281 | Ahu Senem Demiröz, Istanbul, Turkey | | 54 | | | F | Multiple | n.a. | n.a. | n.a. | Composite lymphoma: NMZL and Hodgkin lymphoma |  |
| LYWS-1309 | Stefano Lazzi, Siena, Italy | | 75 | | | F | Multiple | B+ | Mutations in TET2, ESH2, PLCG2, RPS15 and JAK1 | n.a. | MZL | EBV positive |
| LYWS-1432 | Mohammad Hussaini, Tampa, USA | | 66 | | | M | axillary | B+ | n.a. | n.a. | NMZL | Sclerosis, many monocytoid B-cells |
| Abbreviations: AITL: angio-immunoblastic T-cell lymphoma; CLL: chronic lymphocytic leukemia; FDC: follicular dendritic cell; HCL-v: hairy cell leukemia variant; IPSID: immunoproliferative small intestinal disease; MZL: marginal zone lymphoma; NMZL: nodal marginal zone lymphoma; PTCL: peripheral T-cell lymphoma; SDRPL: splenic diffuse red pulp small B-cell lymphoma; SMZL: splenic marginal zone lymphoma; TFH: T follicular helper; VUS: variant of uncertain significance | | | | | | | | | | | | |
